# Microcantilever Displacement Measurement Using a Mechanically Modulated Optical Feedback Interferometer

**DOI:** 10.3390/s16070997

**Published:** 2016-06-29

**Authors:** Francisco J. Azcona, Ajit Jha, Carlos Yáñez, Reza Atashkhooei, Santiago Royo

**Affiliations:** Centre for Sensors, Instruments and Systems Development (CD6)-UPC BarcelonaTech, Rambla St. Nebridi 10, Terrassa E-08222, Spain; ajit.jha@upc.edu (A.J.); carlos.yanez@upc.edu (C.Y.); reza.atashkhooei@upc.edu (R.A.); santiago.royo@upc.edu (S.R.)

**Keywords:** optical feedback interferometry, displacement measurement, nanometric resolution, atomic force microscopy

## Abstract

Microcantilever motion detection is a useful tool for the characterization of the physical, chemical and biological properties of materials. In the past, different approaches have been proposed and tested to enhance the behavior, size and simplicity of microcantilever motion detectors. In this paper, a new approach to measure microcantilever motion with nanometric resolution is presented. The proposed approach is based on the concept of mechanically-modulated optical feedback interferometry, a technique that has shown displacement measurement capabilities well within the nanometric scale and that, due to its size, compactness and low cost, may be a suitable choice for measuring nanometric motions in cantilever-like sensors. It will be shown that the sensor, in its current state of development, is capable of following a cantilever sinusoidal trajectory at different sets of frequencies ranging up to 200 Hz and peak to peak amplitudes up to λ/2 with experimental resolutions in the λ/100 range.

## 1. Introduction

The measurement of physical, chemical and biological properties, especially in the microscopic and submicroscopic scales, typically relies on the use of indirect estimation methods. In the last three decades, cantilevers [1,2,3] and transducers with a mass-spring-like behavior [4] have been used, e.g., for the characterization of micron and sub-micron sample profiles, surface force measurements (e.g., van der Waals, ionic, electrostatic) [5,6], the detection of protein interactions [7] or measuring adhesion forces between cells [8,9]. In all of these cases, the transducer motion is used for retrieving a desired property by relating its behavior to a defined mechanical model with well-known parameters.

In particular, cantilever and microcantilever transducers are interesting indirect measurement tools, since it is possible to attach a different type of tip at the free end of the cantilever, allowing versatility in the materials and analysis to be performed. Furthermore, the mechanical properties of cantilevers are simple enough to be easily estimated using well-known classical methods, such as Euler–Bernoulli [10] or Timoshenko beam theory [11]. Besides theoretical methods, other approaches have been proposed [12,13,14] for the characterization of the cantilever dynamic properties, e.g., the quality factor *Q* and the resonance frequency $f_{r}$, resulting in the possibility of a dynamic analysis of the cantilever motion, such as the ones described in non-contact and tapping mode atomic force microscopy (*AFM*) techniques.

Provided that the cantilever is properly characterized using one of the methods mentioned above, it is only necessary to measure the cantilever motion with enough resolution to provide an estimation of the force applied at the tip of the cantilever and, furthermore, to relate the obtained force to a desired property. To measure cantilever motion, different types of sensors and technologies have been proposed [15]. Typically, optical sensors are preferred over other technologies, since they do not exert an external loading over the cantilever, thus preventing possible bias on the measurement.

The principle of the optical lever technique [16] is one of the most used methods for measuring microcantilever deflection. This technique allows a high sensitivity of the cantilever deflection angle by sensing the position of the laser spot relative to the central position of a four quadrant photodiode. The main problems it presents are the requirement of a metal coated cantilever (which may induce deformations due to the bimetallic effect [17,18]) and a delicate alignment of the three elements involved (laser, cantilever and photodiode).

Classical interferometric methods have also been suggested for measuring cantilever motion. They provide high resolution, usually below 1 Å, at the expense of requiring an expensive and bulky setup, which might prove difficult to align. To partially solve this issue, particularly in the microscale, optical fiber interferometers have been proposed. Nevertheless, the use of optical fibers may induce additional errors in the shape of thermal drifts in low time scales [19], and it will also require special handling to prevent stress during the positioning.

In order to simplify the alignment constraints in cantilever deflection sensing, we propose the use of a sensor based on optical feedback interferometry (*OFI*) [20,21,22]. Sensors based on the OFI technique can be described as compact and self-aligned, thus minimizing alignment issues at least in one degree of freedom while the sensor is kept in a small encapsulation.

Optical feedback interferometry has been previously explored as a method for estimating cantilever deflection. In [23], Ovryn and Andrews measured micrometric scale displacements on a millimetric length cantilever using a phase shifting approach. In this method, a calibration for different amplitudes and frequencies is performed using an electro-optic modulator. After a calibration and a phase unwrapping process in the cases where amplitudes are larger than half-wavelength (λ/2), the net peak amplitude of the deformation is obtained. According to their results, it is possible to achieve similar results to those obtained with holographic interferometry in terms of peak to peak amplitude and main resonance frequency. In [24], Larsson et al. used a vertical cavity surface emitting laser (*VCSEL*) and an external photodiode (*PD*) to characterize the vibration of a polymer micrometric-sized cantilever. In their experiment, the VCSEL is placed at approximately 35 μm from the cantilever. The cantilever is then oscillated by a piezoelectric actuator in a range of 1.2 μm. The OFI signal is then recovered using a voltage junction approach [22], and it is also recovered using an external PD placed above the cantilever. As a result of the motion, a cyclic sinusoidal-like behavior correspondent to OFI is observed. In some sections of the displacement they observed linearity regions with an equivalent slope of 45 mV/nm for the PD measurements. This ratio, however, is only found in small intervals of the measurement (±25 nm), thus requiring locking the measurement region around this point to provide a linear response of the cantilever deflection. In this work, we propose the use of a method based on the differential optical feedback interferometry concept [25], further on referred to as mechanically-modulated OFI. Through this approach, it is possible to obtain resolutions in the order of λ/100. In comparison to the method presented in [23], mechanically-modulated OFI presents a smaller setup, which, due to the decrease in the number of optical elements, reduces the alignment constraints. Furthermore, the complexity of the method is reduced, as the technique depicted in [23] requires at least five intensity measurements. Comparing mechanically-modulated OFI to the method described in [24], the technique presented in this paper removes the need of an external photodiode and increases the measurement range, as the method allows estimating displacements up to an amplitude of λ/2, while the linearity region shown in [24] is limited to λ/8 approximately. The inclusion of a focusing lens also improves the system, as it allows changes of the distance between the cantilever and the sensor, as well as a degree of tuning for the amount of feedback.

In the next section, a brief review of OFI and the mechanically-modulated OFI is presented to enhance the readability of the paper. In Section 2, a complete description of the sensor and the setup used for this work is presented. Furthermore, the calculation methods and the steps followed during the measurement are described. Next, in Section 3, the results obtained are shown. A brief discussion of the results obtained and the strengths and drawbacks of the method proposed are commented on in Section 4.

## 2. Materials and Methods

### 2.1. Mechanically-Modulated Optical Feedback Interferometry

Optical feedback interferometry, also known as self-mixing interferometry (SMI), is a well-known technique capable of measuring changes in the optical path of a laser beam with a typical resolution of (λ/2) for displacement measurements using a fringe counting method (FCM). OFI has been successfully implemented in diverse fields, which range from motion in solid and liquid interfaces [26,27,28] to biological applications [29].

The OFI effect is produced whenever a laser electric field is partially backscattered from an object or particle affected by an optical path change (e.g., displacement). After re-entering the laser cavity, the electric stationary wave and the backscattered wave interact, producing interference phenomena, which is observed as a beat of the laser optical output power (OOP). The OOP can then be retrieved using a photodiode placed in the same package of the laser, or, alternatively, using the voltage variations appearing in the laser junction [22,30].

The OFI process can be modeled using the excess phase equation derived in [20,22,31]:

(1) $$\begin{array}{ccc} \Delta \phi & = & \left(\phi_{f}-\phi_{0}\right)\tau_{e}+C\sin\left(\phi_{f}\tau_{e}+\arctan\alpha \right) \end{array}$$

(2) $$\begin{array}{ccc} P & = & P_{0}\left[1+m\cos\left(\phi_{f}\tau_{e}\right)\right] \end{array}$$

where $\phi_{0}$ is the phase of the free running laser, $\phi_{f}$ is the phase of the laser under feedback, $\tau_{e}$ is the time of flight of the external cavity, *α* the linewidth enhancement factor and *C* the feedback factor [32]. The optical power under feedback *P* can then be represented as the sum of the laser power before feedback $P_{0}$ and the back-reflected optical power, which is described as a proportional part (m≪1) of $P_{0}$, which depends on $\phi_{f}$ and $\tau_{e}$.

The feedback factor *C* is used to discriminate between different working regimes and consequently gives information on the behavior of the OOP AC modulation [22,33]. As the value of *C* increases, a deformation from the original sinusoidal signal is observed until discontinuities appear, generating the well-known sawtooth-like interferometric pattern (C=1). Beyond this point, other effects, such as hysteresis, fringe loss and even chaos, may appear on the signal [22].

For the case of displacement measurements and specially for the implementation of the techniques to be discussed further on, it is generally of interest to work with a C≃1, in order to obtain a high signal to noise ratio (*SNR*) while avoiding potential fringe loss, typical of higher feedback regimes [26]. Working within this regime also allows detecting the target motion direction without ambiguity.

Basic OFI processing methods, such as FCM, are typically limited to a λ/2 resolution. In order to increase the resolution of the technique in displacement measurements, different methods have been reported [22,34,35,36], allowing one to obtain resolutions down to λ/100. In the case of vibrations with amplitudes smaller than λ/2, it has also been shown that it is possible to obtain a responsivity in the range of 4.25 mV/μm and theoretical noise figures in the *p*m/$\sqrt{Hz}$ scale by using the lock-in and phase nulling method described in [37] at the cost of increasing the complexity of the electronic card readout and requiring large distances with respect to the measurement object. Other methods [22] based on an output power calibration have also shown vibration measurement capabilities within the nanometric scale. These methods, however, require the use of other elements, such as electro-optical modulators.

**Figure 1:**
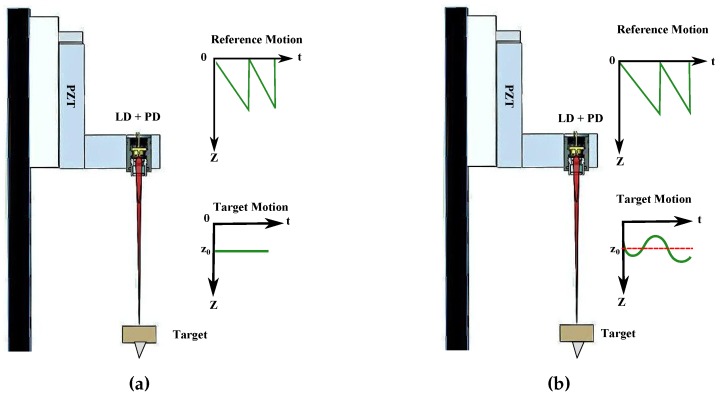
Example of a mechanically-modulated optical feedback interferometry (OFI) setup. (**a**) Reference signal acquired while the target is in a static position with respect to the Y axis; (**b**) The measurement signal is acquired while both the target and the PZT are in motion.

In order to measure within the nanometric scale, we recently proposed the use of differential OFI [25], which has shown practical resolutions in the order of λ/100 in laboratory conditions. The concept of differential OFI, however, can also be applied to single laser diode (*LD*) setups, such as the one shown in Figure 1. For the purpose of clarity, we will refer to this method as mechanically-modulated OFI. In this type of configuration, an LD is pointed at the target and subjected to a reference motion during the whole measurement process. The measurement is divided into two steps. First, as shown in Figure 1a, an OFI signal with C≃1 is acquired for a known piezoelectric (PZT) motion. Next, in a second step, shown in Figure 1b, the measurement signal is acquired while the target undergoes a sub-λ/2 motion, while the PZT keeps the motion used during the reference acquisition.

**Figure 2:**
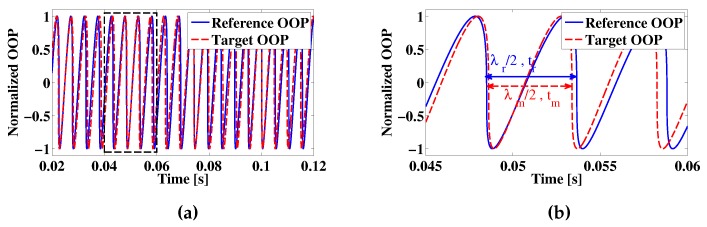
Simulated fringe position change on a measurement signal as a result of a target displacement. The subindex *r* is used for the reference signal and *m* for the measurement signal. (**a**) Comparison between a simulated reference OOP (solid line) and a simulated OOP during target displacement(dashed line); (**b**) Zoom around the region inside of the dashed rectangle in (**a**).

To understand the concept of the technique, consider the effects of setting into motion the displacement stage (e.g., a piezoelectric stage (PZT)) with a peak to peak amplitude $A_{pp}>k\lambda$, where $k\in Z^{+}>2$, in order to create at least 3 measurement points, while the target is kept at rest. As a result of the PZT displacement and given a C≃1, a sawtooth-like pattern will be generated on the OOP with sharp transitions (fringes) generated at each λ/2 displacement. Furthermore, if the reference displacement is linear, the time intervals between two consecutive fringes ($\Delta t_{r}$) should be equal. Assuming no other perturbation is affecting the OFI signal, it is possible to consider the captured data as a reference signal for further measurements. Now, consider that, in addition to the reference displacement, the target starts moving with a peak to peak amplitude $A_{t}\ll \lambda /2$, hence generating the measurement signal. As a result of the target displacement, the time intervals between consecutive fringes in the OOP are no longer constant. An example of this effect is shown in Figure 2, where the dashed line represents the measurement signal. In Figure 2, as well as in all of the following formulas, the sub-index *r* is used to define values related to the reference signal, while the sub-index *m* marks those values related to the measurement signal.

As is shown in [25], it can be proven that for a small displacement Δd, the resolution of the technique can be expressed as:

(3) $$\Delta d=\mp \frac{\lambda_{r}}{2}\frac{\Delta t}{\Delta t_{r}}$$

where $\Delta t=\Delta t_{m}-\Delta t_{r}$ is the difference between the fringe time intervals in the reference and measurement signals and is limited by the sampling rate of the acquisition device and the relative separation of the fringes. In theory, resolutions better than λ/1000 might be achievable, while in practice [25], resolutions in the order of λ/100 have been obtained.

The sampling frequency of the sensor can be modeled as:

(4) $$f_{s}=2\frac{v_{r}}{\lambda_{r}}=4\frac{A_{pp}f_{r}}{\lambda_{r}}$$

where $v_{r}$ is the reference velocity of the linear reference displacement. In order to limit the sensor size and to minimize the speckle effect [38] present on large displacements, it is preferred to use a triangular reference displacement motion with amplitudes in the order of 40 μm or lower. Thus, for a triangular displacement, the sampling frequency is proportional to $A_{pp}$ and the frequency $f_{r}$ of the motion. Due to limitations in mechanical displacement with high linearity, mechanically-modulated OFI is usually limited to work in a low frequency band reaching up to a few kHz.

Although the above described setup could be bulky compared to the original OFI setup, the sensor can be built in a reduced area. Moreover, the sensor’s size may be reduced even further by using an electrical modulation version, such as the one suggested in [39]. For the current implementation, however, the mechanical modulation might present an advantage over the electronic modulation due to its lower dependence on the distance to generate sampling fringes. A more detailed description of the specific methods used in this work to produce the reference signal can be found in Section 2.2.2.

### 2.2. Materials

**Figure 3:**
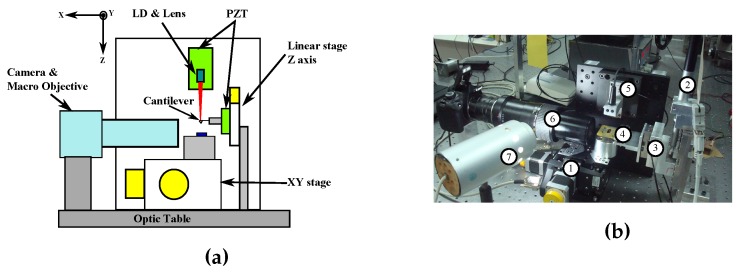
(**a**) Schematic of the mechanically-modulated OFI AFM setup; (**b**) Photograph of the implemented experimental setup: (1) XY translation stage; (2) Z-axis translation stage; (3) piezoelectric vibration displacement stage; (4) cantilever holder; (5) mechanically-modulated OFI sensor; (6) camera with macro objective; (7) white light source for imaging.

To test the capabilities and limitations of the mechanically-modulated OFI sensor, the custom setup shown in Figure 3b was built. As is observed in the schematic shown in Figure 3a, the sensor performs the measurement along the Z axis, while it is shinned at the tip of the cantilever. The description of the setup is divided into three main groups of components: positioning elements, the sensor itself and the digital acquisition equipment.

The mechanical setup consists of 2 PI XY LS-120 stepper motor tables (labeled as (1) in Figure 3b) with a step resolution of 0.2 μm and a maximum travel length of 40 mm, which will be used in later stages to change the relative position between a sample surface and the cantilever probe. A PI M-227.50 translation stage (labeled as (2)) is used to control the Z-axis positioning of the cantilever with a resolution of 50 nm. A PI-LISA piezoelectric (*PZT*) stage (3) with a maximum travel length of 25 μm is used to generate a sinusoidal motion for the cantilever holder (4) and the cantilever. The PZT includes an internal capacitive sensor with a maximum resolution of 2 nm, which will be used in the measurement for comparison purposes. An *AppNano SHOCONA* cantilever with aluminum coating was selected as the target and experimentally characterized yielding an experimental resonance frequency of 45.29 kHz, a quality factor on air of Q=108.70 and a length of L=225 μm.

**Figure 4:**
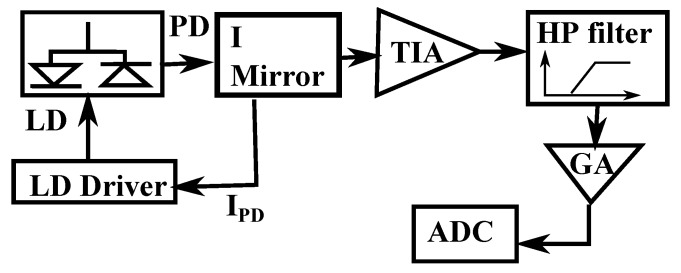
Electronic scheme of the mechanically-modulated OFI analogue acquisition and preconditioning stage. The PD current is passed through a current mirror (I mirror) to prevent loading effects on the transimpedance amplifier stage (TIA).

The sensor (5) is composed of a *Hitachi HL7851G* multi-quantum well Fabry–Perot laser diode with λ=783.5 nm measured using *Instrument System’s SPECTRO 320(D)R5*. The LD is mounted on a Thorlabs *LT230220P-B* collimating tube equipped with a *C230220-B* aspheric lens pair with numerical aperture NA=0.55/0.25. The tube is then attached to another PI-LISA PZT with a maximum travel length of 38 μm using a custom aluminum support, which will be used to oscillate the laser and create the interference fringes. The sensor is placed at an approximate distance of 100 mm from the cantilever. The LD is then connected to a custom-made electronic card, which collects the photodiode current ($I_{PD}$) and sends it to a current mirror (I mirror in Figure 4) allowing us to obtain two duplicates of $I_{PD}$. One of the signals is sent to the LD driver and the second one to a passive transimpedance amplifier (*TIA*). After the conversion to voltage, the signal is subjected to a high pass filter with a cutoff frequency to remove the DC part of the signal. Finally, the signal is subjected to a gain stage (GA), and it is digitized. A block representation of the connection scheme is shown in Figure 4.

The acquisition stage uses a *Tektronix DPO2024B* oscilloscope using one channel to recover the OFI signal and a second channel to retrieve the displacement of the 25 μm LISA PZT. The oscilloscope signal is directly captured in the PC using a custom-made interface developed on VC++ 2010.net. Besides the oscilloscope acquisition, the application controls the parameters of a *Tektronix AFG3022B* signal generator, which controls the PZT drivers and commands the XYZ stage positioning. A *Canon EOS1100D* camera equipped with a *MP-E 65mm f/2.8* macro objective (6) and a white light source (7) is used to provide visual feedback regarding the vertical position of the cantilever.

#### 2.2.1. Scanning and Signal Acquisition

**Figure 5:**
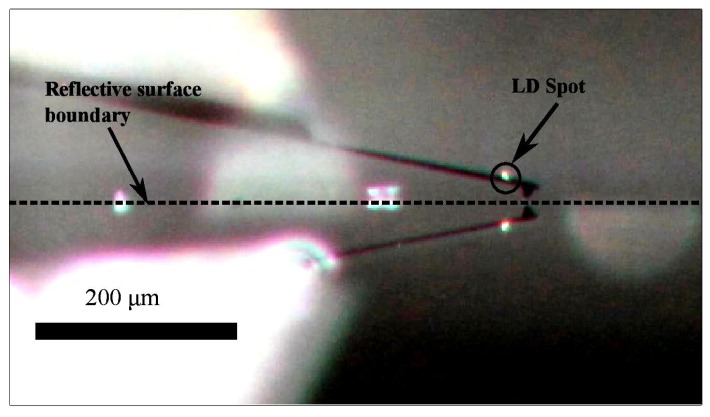
Microcantilever SHOCONA with the reflecting laser spot from the OFI sensor.

Previous to the start of the measurement, a SHOCONA cantilever is placed on a cantilever holder, which is then fixed on a custom mechanical support connected to the PZT, as shown in Figure 3b (3) and (4), then, the LD is focused at the cantilever. The alignment between the cantilever and the laser beam is confirmed by looking at the reflection of the laser spot on the cantilever through the camera, as shown in Figure 5. Once the alignment is confirmed, the XY table is calibrated and set at its origin until a sample is placed on the stage for measurement. After displacing the sample holder and the sample to the measurement coordinates, the cantilever is moved to the required working distance from the sample, and an offline test is performed to check that the OFI signal is working under the desired feedback regime (C≃1). After moving up the cantilever to prevent a collision with the sample, the XY table is moved to the initial scanning position. At this point, it is possible to change the starting scanning position relative to the initial preset coordinate. Next, the cantilever is approached as close as possible to the sample surface. The approach is controlled manually using custom software with the visual aid of the camera.

Once the cantilever and sample are at the initial measurement position, a reference measurement is taken by inducing a triangular mechanical modulation on the OFI sensor. The captured signal corresponds only to the positive ramp of the PZT displacement. After storing the reference measurement, a sinusoidal vibration in the range of 10 to 200 Hz is induced on the cantilever by means of the PZT shown in Figure 3b. The start of the acquisition for the measurement signals is launched using the rising edge of a square transistor-transistor logic (*TTL*) signal that is matched with the start of the positive ramp translation of the PZT, and it is stopped on the falling edge of the TTL signal. After each acquisition, the signals are automatically saved in a PC for further offline signal processing.

After each measurement, the sample is displaced either in the X- or Y-axis in order to test the effects of the raster scanning functionality to be used in further developments. Once the sample is set in the new position, the vibration process is restarted using the parameters previously selected by the user. The process is repeated for the number of points (distance) selected in the software interface.

#### 2.2.2. Signal Processing

As discussed in Section 2.1, the implemented method uses a mechanically-modulated OFI sensor. The reference OOP signal can be obtained in four possible ways: (1) acquiring a reference OFI signal previous to the measurement with an static target; (2) using the piezoelectric internal capacitive sensor to estimate the reference velocity; (3) measuring the mean velocity by detecting the main frequency of the OFI signal spectra given that the measurement process is dynamic; or finally, (4) using the PZT (Figure 3b element (5) capacitive sensor signal to generate a simulated OFI signal at a suitable *C* value. For the results presented below, a reference signal was acquired for each sample set with a static target. In the case of simulations, however, the frequency spectra of the mean velocity method are evaluated, as this is the most suitable implementation in the case that no other type of reference signal is available.

Each method presents different advantages and disadvantages. The first method is preferred over the other noted methods, since it allows one to obtain a better notion of the OFI signal behavior of the laser. This type of acquisition will also reduce the effects of non-linearities, which might occur along the reference motion. Yet, this method requires the use of memory space to store the reference signal, as well as the extra computing cost used for the FCM, which is required to be performed at least once for a set of measurements. The second method reduces the amount of computing resources, as the reference velocity can be calculated directly from the ramp acquired in the PZT sensor. The use of this method may result in a bias, since the velocity is estimated using only the maximum and minimum points of the ramp. The third method reduces the use of memory resources, as the reference velocity is estimated directly from the mean velocity measured with each OFI signal. This method requires the use of an FFT algorithm and also might cause a bias in the measurement similar to the one of the second method. The fourth method is similar to the second one, as it also requires capturing the signal of the PZT capacitive sensor; nonetheless, the computing cost increases as an OFI signal simulation is required. If the laser is well characterized, the resulting bias should be lower than in the second method, as the complete displacement, as well as some of the disturbances suffered by the PZT can be considered.

**Figure 6:**
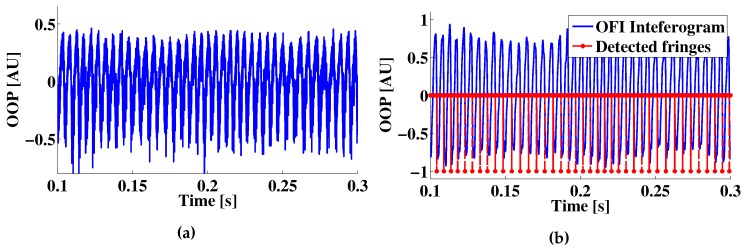
OFI signal fringe detection. (**a**) An example of an experimentally-acquired OFI signal of a ramp-like motion with $v_{r}=76$ μm/s; (**b**) The OFI signal shown in (**a**) after filtering and fringe detection.

After the acquisition, the measurement sample (e.g., Figure 6) is band-pass filtered using an FFT-based filter with cutoff frequencies at $f_{OFI}/5$ and $10\times f_{OFI}$, where $f_{OFI}$ is the main frequency of the OFI signal. Next, the filtered OFI signal is treated with a fringe detection algorithm. For the case of signals presented in this paper, the fringe detection algorithm consists of the differentiation of the OFI signal, after which an initial fringe detection is performed using a threshold comparison method. The threshold is calculated as the RMS of the differentiated signal. After the initial fringe detection, a zero crossing algorithm is applied on the filtered OFI signal, and the detected positions are compared to those in the differentiated signal. This step allows one to remove the possible false positives of the first detection.

After the fringe detection process, the time intervals between consecutive fringes in the measurement and reference signals are calculated, as well as the corresponding velocity given that the distance between two fringes is fixed as λ/2. After obtaining the velocity waveform, the signal is integrated using the cumulative trapezoidal method as described in [25]. In the case of AFM measurement, this process is repeated for each of the acquired signals.

## 3. Results

### 3.1. Simulation

A simulation of a sinusoidal cantilever motion detection using the mechanically-modulated OFI method was performed in order to detect possible limitations on the detection of the sensor. The OFI algorithm solves the excess phase Equation (1) using the Schröeder root finding method and bracketed with the intervals described in [33]. The parameters of the simulation are presented in Table 1.

The chosen parameters were selected to provide a C≃1 (C=0.976) to comply with the expected requirements of the method. A positive ramp-like displacement with a constant velocity $v_{r}=380$ μm/s is chosen as a reference for the OFI signal. All simulated displacement vectors, as well as the corresponding OFI signals are composed by 125 kS to match the length of acquisition vectors in the experimental measurements.

**Figure 7:**
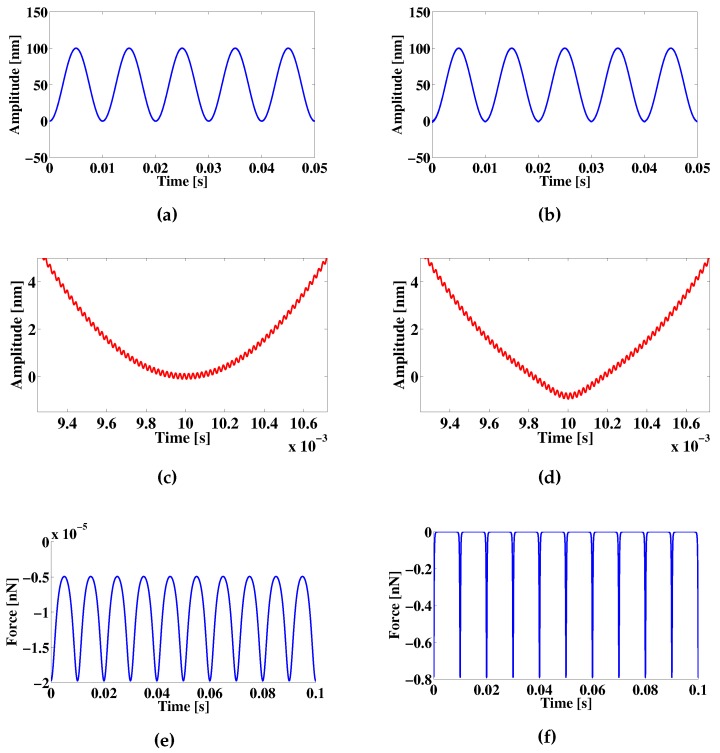
Example of a simulated cantilever tip behavior due to a harmonic motion. (**a**,**b**) Amplitude representation of a harmonic displacement with the tip placed for $d_{z1}$ and $d_{z2}$ respectively; (**c**,**d**) Zoom of the displacement amplitude around the region where the tip is closer to the sample; a deformation of the harmonic motion increases as the minimum tip to sample distance is reduced; (**e**,**f**) Corresponding force effect for $d_{z1}$ and $d_{z2}$.

For the cantilever motion, a harmonic modulation is calculated using as a reference the mass-spring resonator equation:

(5) $$F_{ext}+F_{d}\times sin\left(2\pi ft\right)=m_{eff}\frac{d^{2}x}{dt^{2}}+f_{\nu }\frac{dx}{dt}+k_{c}x$$

where the effective mass $m_{eff}$, damping factor $f_{\nu }$ and stiffness $k_{c}$ are mechanical cantilever parameters relative to the displacement *x* and its time derivatives, $F_{d}$ is the driving force amplitude and $F_{ext}$ accounts for the van der Waals (VdW) force interaction using as reference the Derjaguin-Muller-Toporov (DMT) model. The simulation is tested for two cantilever tips to sample minimum distances $d_{z1}=100$ nm and $d_{z2}=0.50$ nm. The resulting cantilever displacement profiles behave following a harmonic motion with a variation in amplitude when the cantilever tip is closer to the sample, as illustrated in Figure 7. The cantilever displacement is only considered for the cantilever tip in a motion parallel to the beam axis.

**Figure 8:**
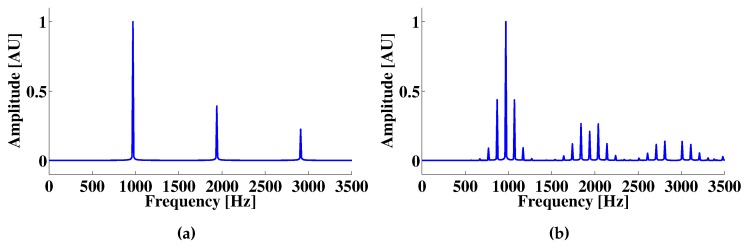
(**a**) Frequency spectra of a simulated mechanically-modulated OFI sensor signal without target motion; (**b**) The effect of the cantilever motion over the frequency spectra; different peaks are observed around the main OFI frequency and its corresponding harmonics; the frequency difference between the main OFI peak and the highest amplitude peak around it is equal to the target vibration frequency.

The resulting OFI signal contains the information of the reference velocity, as well as of the target displacement, as is displayed in Figure 8b by the frequency peaks induced over the spectra due to the Doppler effect. It should also be noted that the normalized amplitude of the frequency peaks caused by the tip motion is dependent on the force resulting in a value of 0.4383 AU for $d_{z1}$ and 0.4389 AU for $d_{z2}$. For the presented simulation, the frequency resolution is limited to 10 Hz. In the case of non-multiple frequencies, the peaks caused by the cantilever vibration become wider, and as in the previous case, their amplitude also changes as a function of their proximity to the simulated sample.

**Figure 9:**
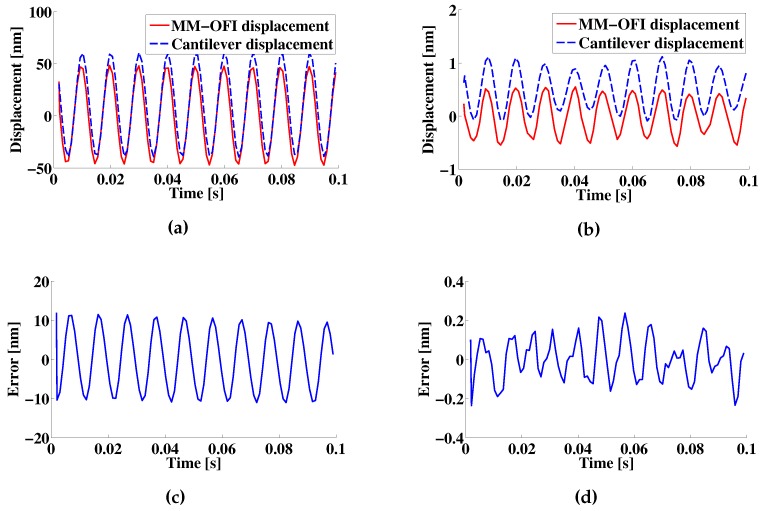
(**a**,**b**) Reconstructed displacements and (**c**,**d**) Reconstruction errors for a 100-nm and 1-nm amplitude displacements. In (**a**), a 10-nm offset is included on the 100 nm displacement for visibility purposes. Similarly, in (**b**), a 1-nm offset is included on the 1-nm displacement for visibility purposes. The legend MM-OFI is used to refer to the mechanically-modulated OFI signal.

After analyzing the spectral effects, the simulated mechanically-modulated OFI signal is subjected to the algorithm discussed in Section 2. In the case of the 100-nm sample distance, the reconstruction follows the simulated cantilever displacement with an RMS error of 7.66 nm and a standard deviation of 7.70 nm, as illustrated in Figure 9. In the case of a distance of 0.50 nm, no significant differences are observed in the reconstruction; however, the RMS error and standard deviation increase respectively in 0.002 nm. The errors in the reconstruction can be related to non-linearities induced by large displacements. This is best observed if we double the velocity of the ramp reference displacement, which results in 3.84 nm and a standard deviation of 3.86 nm, respectively. This is also observed when a 1-nm amplitude displacement is applied as a cantilever motion in the simulation. The resulting RMS error in the reconstruction is reduced to a change in path length of 0.161 nm and a standard deviation of 0.1615 nm.

### 3.2. Experimental Results

**Figure 10:**
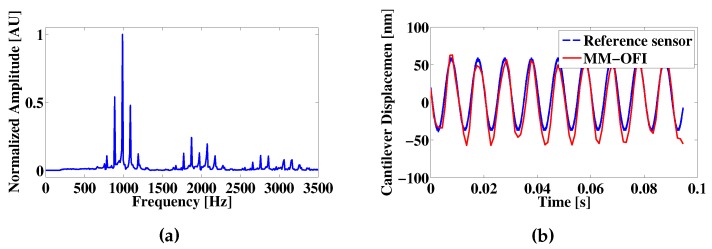
(**a**) Frequency analysis of a mechanically-modulated OFI signal during an experimental cantilever vibrating at 100 Hz with a peak to peak amplitude of 100 nm; (**b**) Comparison between the internal capacitive sensor response of the PI-LISA PZT (dashed line) and the mechanically-modulated OFI sensor (solid line).

On the first test, a set of mechanically-modulated OFI measurements was obtained in regular lab working conditions at a room temperature of 25 ^∘^C. A 100-nm peak to peak oscillation was induced on the cantilever, and the signals were retrieved following the steps described in Section 2, resulting in the waveform shown in Figure 10. Reconstruction errors can be attributed to three major sources: (1) the linearity of the reconstruction algorithm; (2) mechanical fixation issues; and (3) mechanical noise caused by acoustic and mechanic vibration of the building itself. Low SNR signals may also induce errors because of the increased difficulty to detect the position of the OFI signal fringes. This effect, however, is limited for the measurements presented in this work, as the sensor is calibrated to work close to C=1.

For the case presented, the difference between the PZT sensor response and the mechanically-modulated OFI measurement can be explained by mechanical fixation issues between the PI-LISA PZT and the cantilever holder and by possible linearity issues in the reconstruction method. Another possible explanation to the difference might be the detection of surface forces over-imposed on the reference displacement as they are within the 20 to 30-nm range, which would represent forces in the range of 0.3 nN to 18 nN. It should be noted that the frequency spectra displayed in Figure 10a show two peaks at 886 Hz and 1088 Hz from the expected 987-Hz frequency. The difference between the central frequency and any of these peaks corresponds to the main driving frequency of the sinusoidal modulation (100 Hz). These peaks also appear at the second and third harmonic of the OFI spectra (1975 and 2962 Hz) with smaller decay than those corresponding to the OFI signal.

**Figure 11:**
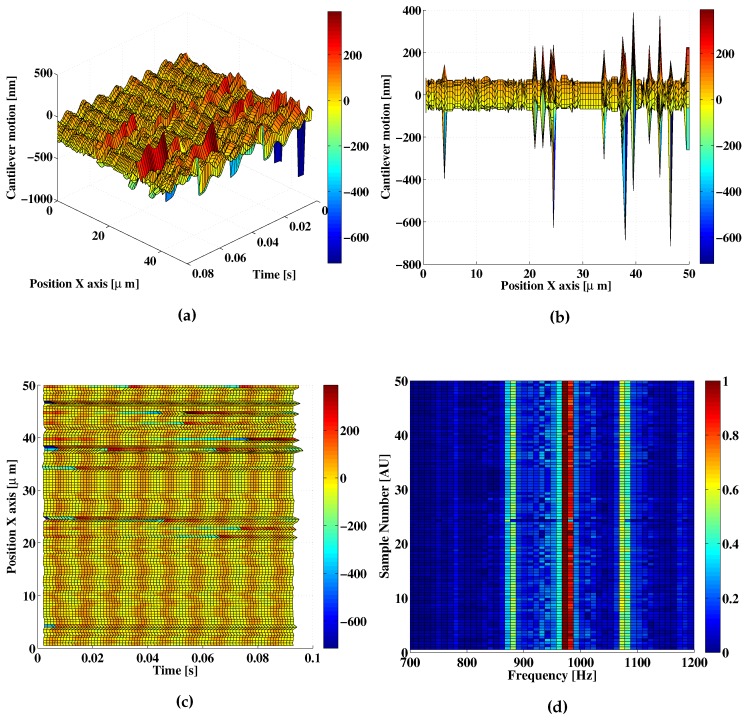
Example of an experimental A=100-nm cantilever displacement reconstruction under uncontrolled acoustic and mechanical noise conditions. The displacement is shown in different projections: (**a**) 3D; (**b**) top and (**c**) lateral view; (**d**) The top view of the Fourier spectra around the main harmonic of the OFI measurement signal for each of the points acquired in the X-axis.

In order to remove some of the issues observed in the previous measurement, the holder grip mechanism was modified increasing the rigidity of the mechanical structure. After a new calibration process, a new set of measurements was acquired under normal laboratory conditions, which include the presence of random mechanical noise and acoustic vibrations. An example of signals acquired for a 50-μm displacement in the X-axis with steps of 0.5 μm is presented in Figure 11. As is observed, there exist different punctual events, which cause errors in the reconstructed measurement, in some cases with amplitudes circa *λ*, as shown in Figure 11b. In most cases, the measurement holds the expected 100-nm amplitude value, as observed in Figure 11c. This stability in the detected amplitude and frequency is also confirmed in the representation of the Fourier spectra for each of the OFI acquired measurements (Figure 11d). The spectra do not show any discernible variations in the amplitude of the signal (which can be obtained by multiplying by λ/4 the normalized amplitude) showing a mean value of 92 nm with a standard deviation of 8 nm for 100 measurements. This, again, has good agreement with the expected capacitive sensor response.

**Figure 12:**
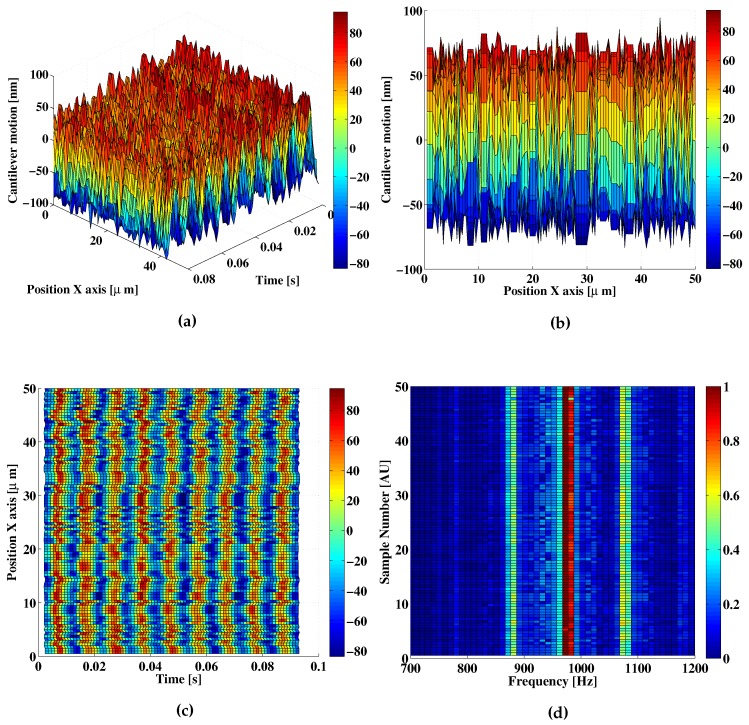
Example of an experimental A=100-nm cantilever displacement reconstruction under low acoustic noise conditions. The displacement is shown in different projections: (**a**) 3D; (**b**) Top and (**c**) Lateral view; (**d**) Top view of the Fourier spectra around the main harmonic of the OFI measurement signal for each of the points acquired in the X-axis.

A second set of measurements was acquired under more controlled conditions. In this case the system was automatized to perform the measurements automatically while there was no one on the room. In principle, this reduced possible sources of acoustic noise during the measurement, and also, sources of mechanical noise induced by footsteps in the room. Again, signals were acquired for a 50 μm displacement in the X axis with steps of 0.5 μm as presented in Figure 12. As it is shown in Figure 12a,c, no spikes of amplitude larger than 100 nm are visible. A random variation on the detected amplitude is observed in Figure 12b, leading us to believe that the proximity to the sample surface may be having an effect on the measurement. These variations, nonetheless, cannot be quantified at the moment. In most cases, however, the measurement holds the expected 100 nm amplitude value as observed in Figure 12c. The stability in the detected amplitude and frequency is also confirmed in the representation of the Fourier spectra for each of the OFI acquired measurements (Figure 12d). The Fourier spectra of the signals shows a mean value of 92 nm with a standard deviation of 8 nm for 100 measurements which again is in good agreement with the results of the capacitive sensor.

The off-line computing time for each signal containing takes in average 0.695 s, which shows feasibility for an on-line signal processing using the mechanically modulated OFI method.

## 4. Discussion

The results obtained in the previous section show the feasibility of a mechanically-modulated OFI sensor for detecting a cantilever sinusoidal motion with peak to peak amplitudes going from 100 to 200 nm. Previous results with the technique [25] show that a practical resolution of 3 nm can be reached, thus allowing the capture of 10-nm amplitudes with sufficient detail.

By means of simulation, it has also been shown that it is possible to reproduce the displacement of lower figures, such as is the case of the 1-nm displacement shown in Figure 9, since the linearity of the sensor is increased for smaller changes. Similar results are also shown in [23], where it is observed that the OFI readings behave linearly for small changes. Nevertheless, attaining such figures seems difficult to reach in practice due to the several sources of noise that might affect either the target or the sensor itself in its present configuration. In particular, it has been observed that the system is extremely sensitive to mechanical and acoustic fluctuations in the room. Therefore, it is required to increase the isolation of the system with the room acoustic conditions, which might be achieved by casing the system in a sound-proof box. External vibrations can be reduced by setting the system on an absorbent material (e.g., marble), which would reduce the amount of vibrations transmitted from the floor into the instrument. Another possibility would be to work on vibration amplitudes with frequencies larger than 200 Hz to reduce possible vibrations inherit to the building structure. In terms of mechanical noise reduction, it is also possible to change the measurement direction towards the X- or Y-axis. This, however, may introduce an issue with sample handling in the case of liquid-based media. In terms of electrical noise, it has been observed that the system is capable of obtaining low errors if the SNR of the signal is larger that 10 dB; for lower values, the signal tends to degrade, reducing the fringe visibility and, thus, affecting the fringe occurrence time detection. For SNRs above 10 dB, detected RMS errors on the signal typically stay below λ/100. The signals presented in this work presented a minimum SNR in the order of 20 dB.

As mentioned before, the normalized Fourier spectra of the mechanically-modulated OFI signal also contain practical information of the displacement, as it can be used to detect the information of the mean velocity used to generate the reference signal and also to compute the main frequency of the sample displacement in the case of the harmonic motion. It should also be noted that for the case where the cantilever was subjected to forces in an order lower than 1 nN, a change on the peak amplitude at the corresponding Doppler frequency shift with a value of 0.0006 units was detected. In principle, this variation is negligible; however, it should be expected that as the sample frequency is increased and reaches values closer to the cantilever resonance frequency, the amplitude will be amplified.

It is also to be noticed that this work focuses on measurements at low frequencies compared to typical measurements in the area that typically work over the kHz range. It is stressed that the work described in this paper is to prove the feasibility of the mechanically-modulated OFI method on the detection of microcantilever displacement in the nanometric scale. The measurements were performed taking into account the limitations of the mechanical systems used to provide both the reference modulation and the cantilever modulation. While working within the selected frequency range can be partially the cause of the noise induced over the measurement, the obtained results show that, nonetheless, it is possible to retrieve a cantilever displacement with nanometric amplitudes.

## 5. Conclusions

In this paper, we have shown the feasibility of using mechanically-modulated OFI-based sensors for the measurement of microcantilever motion. To the extent of our knowledge, this is the first time that this method has been applied to the measurement of microcantilever motions. The technique, as it is described, is able to recover the motion of a commercial microcantilever usually employed in AFM systems, suggesting that the sensor should be able to detect the motions of micrometric cantilevers, as long as the displacement is within the resolution of the device. While experimental conditions and available materials did not allow testing the method for amplitudes below 100 nm, larger amplitude motions show good agreement with the theory and simulations presented. Furthermore, the frequency analysis shows good correspondence with the theory showing the appearance of secondary peaks in the Fourier spectra with the correct amplitude relative to the normalized amplitude of the principal OFI harmonic and with a shift in frequency corresponding to the main frequency of the cantilever motion.

There exist several factors that require further work before creating a practical mechanically-modulated OFI sensor for cantilever motion measuring. First of all, a better mechanical noise isolation system is required since, as observed in Figure 11, some noise sources may drastically hamper the results on the measurements. Some possible solutions to achieve this isolation are described in Section 4. Furthermore, the change of some conditions on the tip holder and the tip itself may benefit the measurement. These changes include reducing the angle between the tip and the horizontal plane and removing the aluminum coating on the cantilever. In the first case, a reduction on the possible fringe detection errors during the signal processing caused by the angle of the cantilever is to be expected. In the second case, the aluminum coating of the cantilever can cause issues due to the bimetallic effect, which introduces strain on the cantilever, causing some degree of bending due to the difference of thermal expansion coefficients between the reflective and substrate materials of the cantilever. This has also been remarked by other authors, e.g., in [18,23], as an issue with classic cantilevers. Even more problematic for the OFI case is the large reflectivity of aluminum, which may change the OFI working point to a strong feedback regime. In the measurements proposed above, a small amount of de-focusing has allowed removing the issue at the cost of having a larger spot size and lower SNR, which also may limit the accuracy of the method.

Towards a profile reconstruction algorithm, further work is required to perform a complete interpretation of the acquired data. For these purposes, a tip force characterization system needs to be included in the processing. The method may largely benefit from the use of other types of OFI setups, such as the one discussed in [39], as they work closer to the bandwidth frequency of more classical commercial instruments. It is important to remark that in its current setup, the sensor is limited by the mechanical modulation imposed by a PZT, thus limiting the reference working frequency as a function of the displacement amplitude. In principle, the sensor described in this paper should be able to measure displacements with frequencies in the order of 1 to 2 kHz with high definition and up to 10 kHz with a lower definition. Further steps will also include the reconstruction of well-controlled sample surfaces in order to clarify the limits on the vertical and lateral resolution of the system.

## Figures and Tables

**Table 1:** Parameters for cantilever displacement simulation.

Parameter	Value	Units
Wavelength (*λ*)	783.50	nm
Reference velocity ($v_{r}$)	380.00	μm/s
Feedback attenuation (*ϵ*)	0.06	
Feedback factor (*C*)	0.98	
Distance to cantilever (*D*)	100.00	mm
Cantilever displacement (ΔD)	100.00	nm
Cantilever frequency (*f*)	100.00	Hz
Cantilever reflectance ($R_{e}$)	0.90	
Distance sample to cantilever 1 ($d_{z1}$)	100.00	nm
Distance sample to cantilever 2 ($d_{z2}$)	0.50	nm
Cantilever stiffness ($k_{c})$	0.95	N/m
Cantilever quality factor ($Q_{c})$	108.70	
Cantilever resonance frequency ($f_{rc})$	45.29	kHz
